# Effect of anti-CD52 antibody alemtuzumab on *ex-vivo *culture of umbilical cord blood stem cells

**DOI:** 10.1186/1756-8722-1-19

**Published:** 2008-10-23

**Authors:** Che K Lim, Li Sun, Qi Feng, Ping Law, Wei T Chua, Shy N Lim, William YK Hwang

**Affiliations:** 1 Department of Clinical Research, Singapore General Hospital, Outram Road, Singapore; 2 Department of Hematology, Singapore General Hospital, Outram Road, Singapore; 3 Stemcyte, Covina, CA, USA; 4 Nanyang Technological University, 50 Nanyang Avenue, Singapore; 5 Duke- NUS Graduate Medical School Singapore, Jalan Bukit Merah, Singapore; 6 Singapore Cord Blood Bank, 100 Bukit Timah Road, Singapore

## Abstract

**Background:**

Excessive maturation of hematopoietic cells leads to a reduction of long-term proliferative capability during cord blood (CB) expansion. In this study, we report the effects of anit-CD52 (Alemtuzumab, Campath) on both short- and long-term *ex vivo *expansion of CB hematopoietic stem cells (HSC) by evaluating the potential role of Alemtuzumab in preserving the repopulating capability in CB HSC and nonlymphoid progenitors.

**Methods:**

*Ex vivo *expansion experiments were carried out using freshly purified CB CD34^+ ^cells in StemSpan™ SFEM medium in the presence of stem cell factor, Flt3-Ligand and thrombopoietin at 50 ng/ml. Alemtuzumab (10 μg/ml) was used to deplete CD52^+ ^cells during the cultures. Flow cytometry was used to monitor CB HSC and their differentiation. Colony forming unit (CFU) assays and long term culture-initiating cell (LTC-IC) assays were performed on cells obtained from day 0 (before culture) and day 14 after cultures. Secondary cultures was performed using CD34^+ ^cells isolated at 35 days from primary cultures and further cultured in StemSpan™ SFEM medium for another 14 days to confirm the long term effect of alemtuzumab in liquid cultures.

**Results:**

Compared to cytokines alone, addition of alemtuzumab resulted in a significant increase in total nucleated cells, absolute CD34^+ ^cells, myeloid and megakaryocytic progenitors, multi-lineage and myeloid CFU and LTC-IC.

**Conclusion:**

The results from current study suggested that the use of alemtuzumab for *ex vivo *expansion of CBHSC maybe advantageous. Our findings may improve current technologies for CBHSC expansion and increase the availability of CB units for transplantation. However, *in vivo *studies using animal models are likely needed in further studies to test the hematopoietic effects using such expanded CB products.

## Background

Umbilical cord blood transplantation (CBT) is now an established means of hematopoietic stem cell (HSC) transplantation for patients with a variety of malignant and non-malignant disorders [1-3]. As the results of partially matched CBT are similar to those of fully matched unrelated bone marrow transplantation, there has been a significant increase in CBT for patients in need of transplantation with matched unrelated allogeneic HSC. This, coupled with the immediate availability of cord blood units (CBU), has made cord blood an ideal source for pediatric HSC transplantation [4-8].

Due to the low number of HSC in each CBU, double unit CBT has been attempted and the results show that the two CBU have an additive effect, resulting in engraftment times equivalent to a single unit CBU with the cumulative cell dose of both the units [9-12]. However, while this treatment modality has increased cord blood usage in adult patients, many patients remain ineligible, as the total cell dose of two CBU may still be insufficient for many adult patients. In recent years, *ex vivo *expansion of HSC has been used as another approach for obtaining sufficient CBHSC from a single CBU in order to obtain adequate repopulating HSC from a single CBU. However, despite considerable research, *ex vivo *expansion of CB HSC has not definitively resulted in improved clinical outcomes in CBT [13-15], and failure or delayed hematopoietic engraftment was encountered in some earlier attempts using *ex vivo *expanded CB products, despite of satisfactory and impressive results from preclinical *ex vivo *experiments [16-18]. Stiff and colleagues have successfully transplanted patients with small aliquots of autologous bone marrow (BM, median volume = 36.7 ml with lowest volume = 13 ml) in Aastrom/Replicell stromal-bases close system in serum-containing medium using GM-SCF-IL-3 fusion protein, Flt3-L and erythropoietin as sole-source of HSC in 19 patients with breast cancer. Long-term hematopoietic reconstitution was achieved without an increase in infection or late graft failure for up to 8 years [14,19]. Rice and colleagues reported that the response to short-term cytokine exposure in different CB hematopoietic cell populations was mainly from the mature cell populations rather than from the stem cell population [20]. Their observation at least partially explained the pitfalls in clinical-scale transplantation using *ex vivo *CB products. To overcome this problem, new approaches for ex vivo expansion need to be developed, aiming at expansion of the "stem" cell population in cord blood and improving the long-term hematopoietic reconstitution in cord blood transplantation [14,21].

CD52 is a phosphatidylinositol-linked, 12-amino acid leukocyte differentiation antigen abundantly expressed on the surface of activated lymphocytes, monocytes, macrophages, monocyte-derived dendritic cells and endothelial cells [22-25]. Alemtuzumab, a humanized anti-CD52 monoclonal antibody, was approved by FDA in 2001 for the clinical administration in patients with chronic lymphoblastic leukemia (CLL). Alemtuzumab results in rapid clearance of CD52^+ ^cells by complement-mediated target cell lysis and antibody mediated cellular toxicity [26,27]. Alemtuzumab is now commonly used for the treatment of lymphoid malignancy, such as chronic lymphoid leukemia (CLL) [28]. Previous publications show that alemtuzumab could also enhance megakaryopoiesis [22,29]. Based on these earlier observations, we postulated that depletion of CD52^+ ^cells with alemtuzumab in *ex vivo *expansion experiments of CBU may lead to a higher percentage of CD34^+ ^cells through depletion of more mature CD52+ hematopoietic progenitors. Hence, we embarked on this study to evaluate the potential role of Alemtuzumab in preserving primitive CB HSC during *ex vivo *cord blood expansion.

## Results

### Evaluation of the quality of CB CD34+ hematopoietic progenitors and stem cells

**Figure 1:**
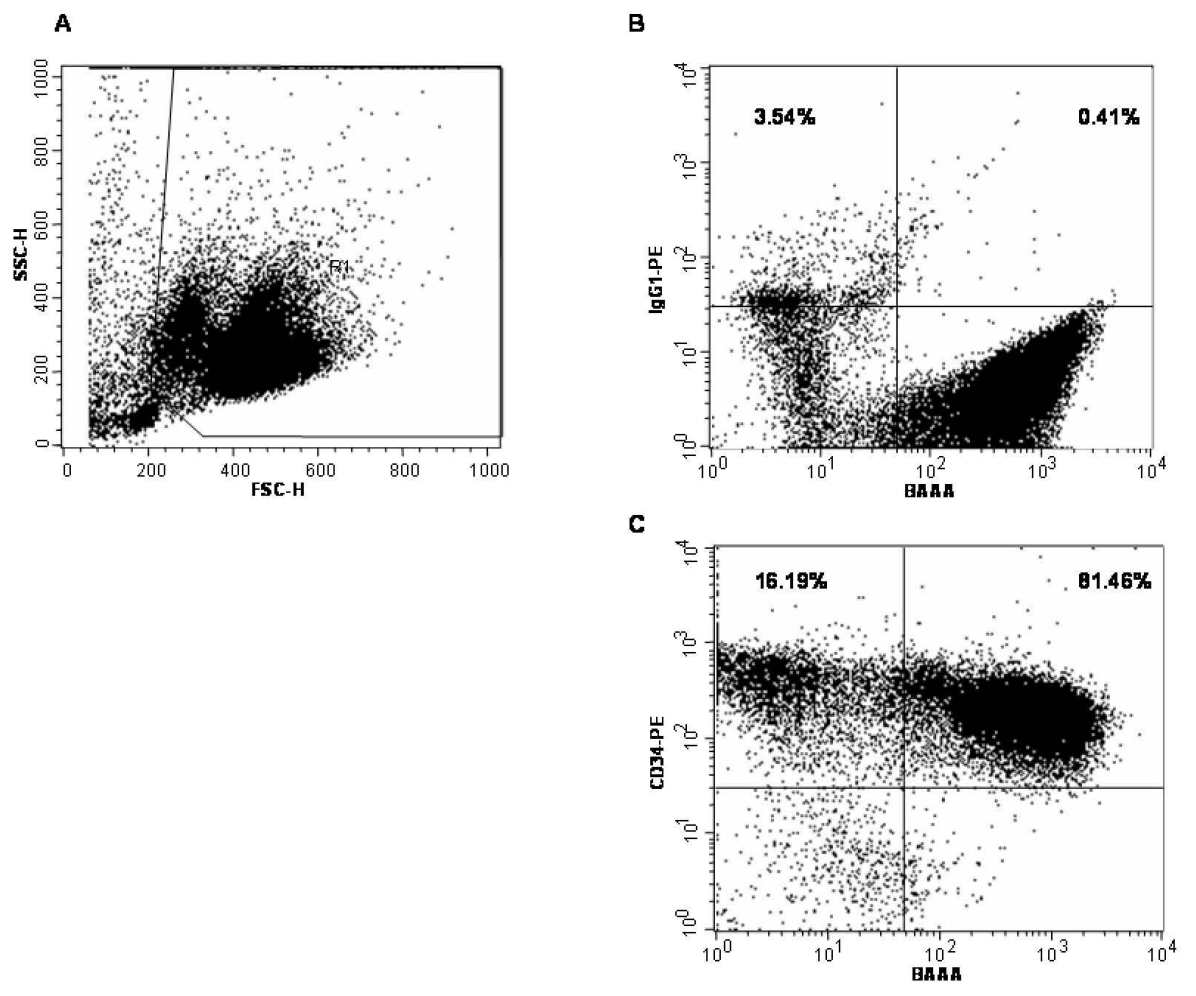
**CD34 selection efficiency by aldefluor assay and flow-cytometry assay**. After CD34 selection, cells were stained with CD34-PE and Aldefluor. CD34+ stem cells were selected according to forward scatter (FSC) and side scatter (SSC) properties using the gated region R1 to remove cell debris, residual platelet and red cell contamination. Figures were representative of 6 separate experiments. A: Phenotype of day 0 CD34^+ ^selected cells that initiate the culture. The percentages of cells positive for the CD34-PE and Bodipy-aminoacetate (BAAA) were obtained from gates R1. B: Isotype expression pattern for IgG-PE and BAAA. C: Expression profile of stem cells co-staining with CD34-PE and BAAA. As shown in the Fig, based on R1 gating, 81.05% cells were both CD34 positive and BAAA brightly stained, 12.65% cells were CD34 positive but BAAA negative. 86.50% of the CD34+ cells were brightly stained with BAAA.

The purity of CD34+ cells after immunomagnetic separation was 85.72 ± 5.98% (n = 6). The result was confirmed by Aldeflour staining (Figure 1); 81.05% cells were both CD34^+ ^and Bodipy-aminoacetate (BAAA) brightly stained, 12.65% CD34^+^cells did not express BAAA activity. In total, there were 86.50% cells brightly expressing BAAA in CD34-enriched cell population. The data are consistent with previous reports [30-32] and provides secondary confirmation of the cell selection efficiency and that primitive and naïve hematopoietic progenitors were present within our initiating cells.

### Effect of alemtuzumab on total nucleated cells (TNCs) and CB CD34^+ ^cells

**Figure 2:**
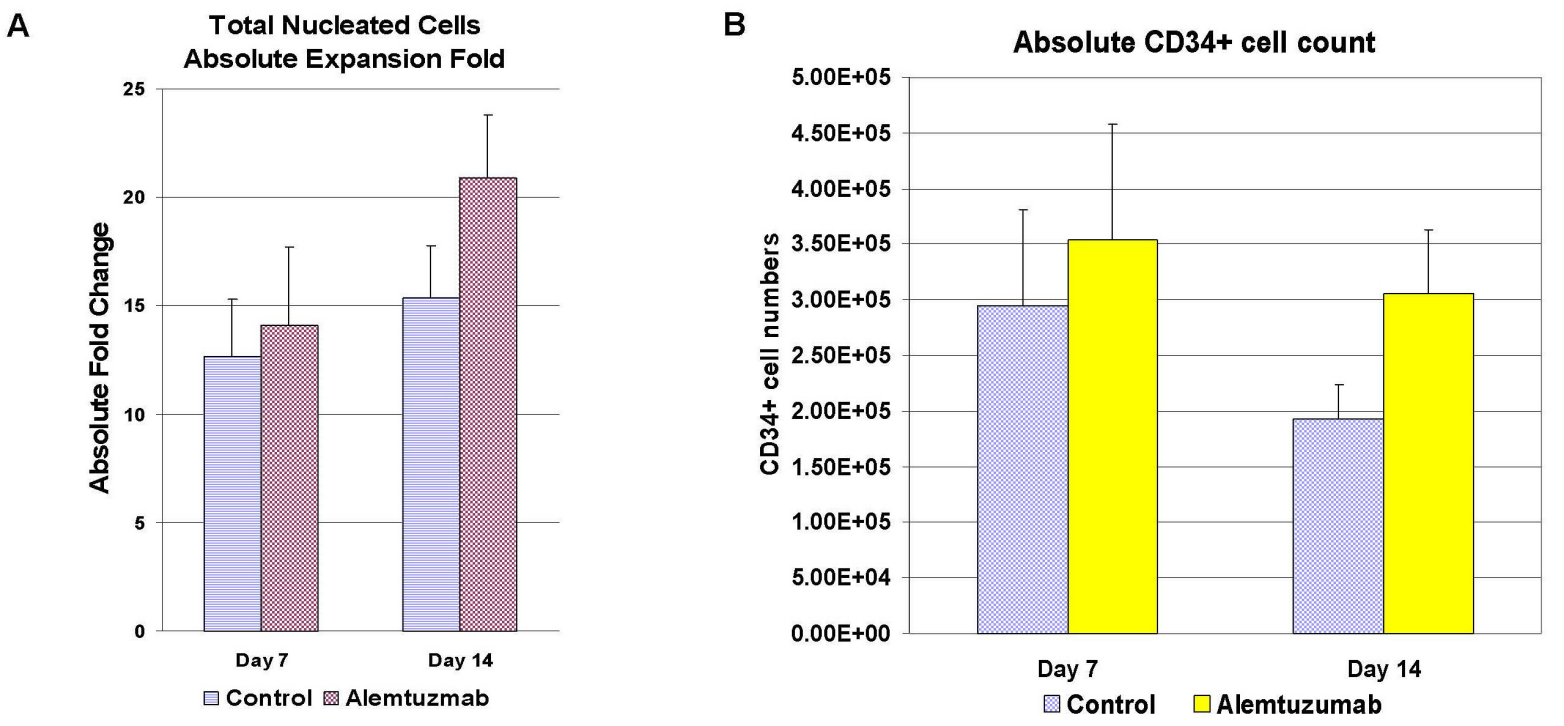
**Effects of alemtuzumab on total nucleated cells and CD34^+ ^cells**. 100,000 CD34+ cells were cultured in 2 ml StemSpan™ SFEM Medium supplemented with 10% of healthy human serum, 50 ng/ml of stem cell factor, TPO and Flt3 Ligand. 10 μg/ml of alemtuzumab was added to test the alemtuzumab's effects on HSC expansion. Cultures were maintained at 37°C with humidified air containing 5% CO_2 _for 5 weeks. A small fraction of the cultures were harvested at day 7, 14 to test absolute total nucleated cell expansion. (A), CD34^+ ^cell percentages (B), Absolute CD34^+ ^cell numbers in control and alemtuzumab treatment groups. Results are representative of 6 separate experiments from different fresh cord blood samples.

As shown in Figure 2A, TNCs were expanded 20.90 ± 2.89 fold on day 14 with alemtuzumab, which was 1.36-fold more than control (15.33 ± 2.45, p < 0.05). The absolute CD34+ cell count (Figure 2B) was 3.06 × 10^5 ^± 0.58 × 10^5^, in cultures with alemtuzumab, compared to control (1.93 × 10^5 ^± 0.31 × 10^5^. Compared to control cultures, the absolute CD34^+ ^cell numbers were 58.55% higher with alemtuzumab added in the cultures (N = 6, p < 0.05).

### Effect of alemtuzumab on lymphoid, megakaryocytic and myeloid cells

As shown on Table 1, the addition of alemtuzumab resulted in a 98.48% reduction in the proportion of absolute CD52^+ ^cells (0.02 × 10^5 ^± 0.02 × 10^5 ^versus 1.32 × 10^5 ^± 0.44 × 10^5 ^in controls; N = 6 p < 0.05) and 96.36% reduction in the proportion of CD90^+ ^lymphoid progenitors (0.02 × 10^5 ^± 0.01 × 10^5 ^versus 0.55 × 10^5 ^± 0.24 × 10^5 ^in controls; N = 6 p < 0.05). Mature lymphocyte markers, such as CD3 and CD19 were also tested, but as the current cytokine combination did not favor lymphoid cell proliferation, the percentage was too low (<0.3%) to demonstrate any potential effects on the addition of alemtuzumab. On the other hand, in the presence of alemtuzumab, more expansion of CD13^+ ^myeloid progenitors (44.35%) was observed compared to controls. The expression of glycophorin A appeared to be marginally enhanced by alemtuzumab, but the difference was not statistically significant.

**Table 1:** Effects of alemtuzumab on lineage differentiation during *ex vivo *expansion of CB stem cells

Cell Surface Markers	Expression Level			
	
	Control Cultures		Cultures with alemtuzumab	
	Percentage ± SD	Absolute Cell Count (× 10^5^)	Percentage ± SD	Absolute Cell Count (× 10^5^)

CD52	8.63 ± 2.49	1.32 ± 0.44	0.10 ± 0.08	0.02 ± 0.02
CD90	3.58 ± 1.46	0.55 ± 0.24	0.10 ± 0.03	0.02 ± 0.01
CD14	23.01 ± 3.73	3.53 ± 0.80	17.71 ± 3.18	3.70 ± 0.84
Glycophorin-A	0.57 ± 0.33	0.09 ± 0.05	0.71 ± 0.48	0.15 ± 0.10
CD13	84.86 ± 4.27	13.01 ± 2.18	89.85 ± 1.88	18.78 ± 2.63
CD41	19.82 ± 1.12	3.04 ± 0.52	22.63 ± 1.32	4.73 ± 0.71

**Phenotype of day-14 *ex vivo *expansion cultures: **Liquid cultures for 14 day ex vivo expansion were established with 100,000 CD34^+ ^cells in 50 ng/ml of SCF, TPO and Flt-3 Ligand. The results of six separate experiments are expressed in terms of different surface marker expression levels on day 14.

### Effect of alemtuzumab on megakaryocytes

**Figure 3:**
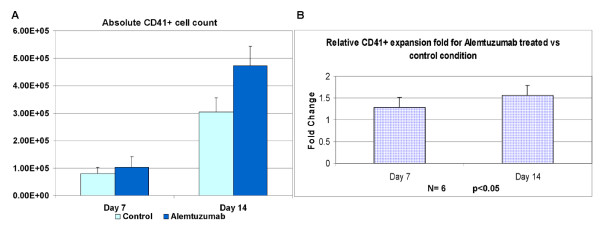
**Effect of alemtuzumab on megakaryocytes**. Cells were cultured in the same condition as described in Fig 2 and harvested at Day 7 and Day 14 for analysis. A: Absolute CD41^+ ^cell numbers. B: Relative CD41^+ ^cell expansion with Alemtuzumab versus control. (N = 6, p < 0.05).

As shown in Figure 3A, there was 28.75% greater expansion of CD41+ megakaryocytes in the presence of alemtuzumab on day 7 cultures (1.03 × 10^5 ^± 0.39 × 10^5 ^with alemtuzumab vs 0.80 × 10^5 ^± 0.23 × 10^5 ^without; N = 6, p < 0.05) and 55.59% greater expansion on day 14 cultures (4.73 × 10^5 ^± 0.71 × 10^5 ^with alemtuzumab vs 3.04 × 10^5 ^± 0.52 × 10^5 ^without; N = 6, p < 0.05). These observations are consistent with previous reports, which show that Alemtuzumab enhances megakaryopoiesis [22,29].

### Colony Forming Unit (CFU) assay

**Figure 4:**
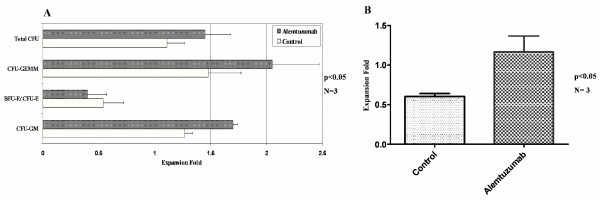
**CFU and LTC- IC expansion fold under different culture conditions**. A: CFU expansion fold under different culture conditions: After 14 days culture, cells were harvested from different culture conditions and put to Methocult H4435 medium for another 2 weeks. CFU-GM, BFU-E/CFU-E and CFU-GEMM were counted according to their morphologic characters. Comparison of absolute CFU (total CFU together with three different types of CFU) expansion fold after 14 days culture with or without alemtuzumab treatment is shown. The results of three separate experiments performed in duplicate are expressed in term of the CFU expansion fold on day 14 compared to day 0 initiating cells. B: LTC-IC expansion fold under different culture conditions: LTC-IC was performed on cells before and after 14 days of culture and, in the absence or presence of alemtuzumab. Results are from 3 separate experiments from performed in triplicate on separate fresh cord blood samples.

CFU-GEMM and CFU-GM were significantly improved with 14 days of alemtuzumab expansion culture. As shown in Figure 4A, CFU-GEMM was expanded 2.05 ± 0.42 fold with alemtuzumab compared to 1.48 ± 0.29 fold in control (p < 0.05). CFU-GM was expanded 1.70 ± 0.04 and 1.27 ± 0.07 fold with or without alemtuzumab treatment respectively. Erythroid colonies (BFU-E/CFU-E) were nearly 2-fold decreased compared to initial cells in both conditions (0.4 ± 0.17 vs. 0.54 ± 0.18 fold expansion with or without alemtuzumab, respectively), which partially offset the total CFU (CFU-GM + BFU-E/CFU-E + CFU-GEMM) expansion (1.45 ± 0.23-fold in alemtuzumab treatment groups, p < 0.05 vs. 1.11 ± 0.16 in control, p < 0.05, compared to initiate cells). To further demonstrate the effect of alemtuzumab on expansion, relative CFU expansion fold was calculated (Fig 3B). Expansion cultures carried out in the presence of alemtuzumab resulted in 1.40 ± 0.06, 1.34 ± 0.04 and 1.31 ± 0.04 fold more expansion in CFU-GEMM, CFU-GM and total CFU respectively (p < 0.05).

### Long-term culture-initiating cell (LTC-IC) assay

LTC-IC was performed on cells before and after 14 days of culture and, in the absence of alemtuzumab, there was a 53% ~75% decrease in LTC-IC compared to initial CD34^+ ^cells (prior to culture). In the presence of alemtuzumab, LTC-IC was sustained at 1.17 ± 0.20 compared to the initial cells (Figure 4B). Thus, addition of alemtuzumab into the cultures resulted in 1.45- to 2.25- (mean 1.95 ± 0.44) fold greater numbers of LTC-IC compared to controls (p < 0.05).

### Secondary cultures of CD34^+ ^cells

**Figure 5:**
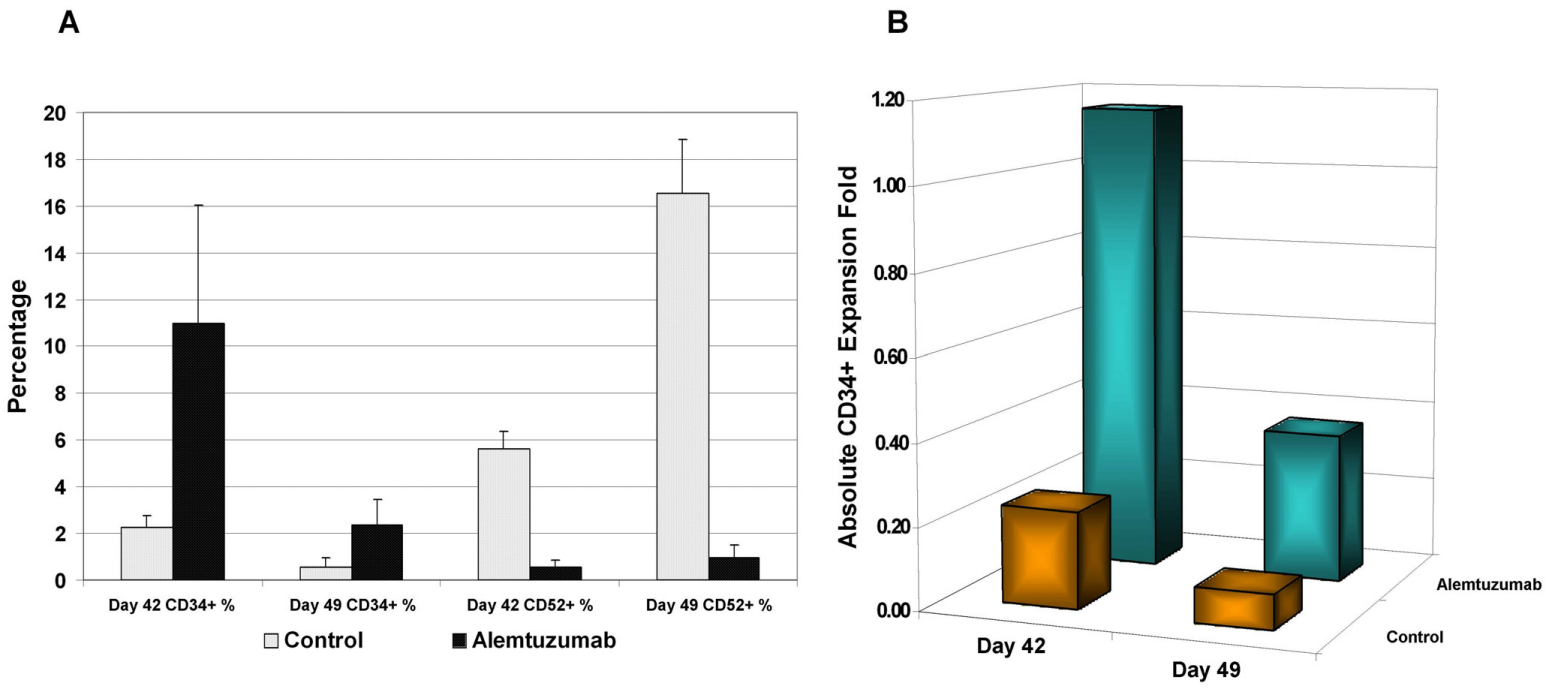
**Alemtuzumab long-term efforts on CD34^+ ^cells secondary culture**. CD34^+ ^cells were isolated from day 35 cultures and put into secondary culture with the original cytokine combination (50 ug/ml SCF, TPO and Flt-3 Ligand) and 10% human serum, with or without 10 μg/ml alemtuzumab (control group). After a further 7 to 14 days of culture, cells were harvested to analyze CD34 and CD52 expression profiles. A: Expression profiles of CD34 and CD52 at Day 7 and Day 14 of secondary cultures. B: Absolute expansion fold of CD34+ cells of secondary cultures. The absolute expansion fold is calculated by the absolute CD34+ cells number as compare to secondary culture initiate CD34+ numbers. The results are from three separate experiments.

Secondary cultures was performed using CD34+ cell isolated from day 35 of *ex vivo *expansion cultures to confirm the long-term growth of CD34+ cell in liquid cultures. The purity of CD34^+ ^cells was 85.00%, similar to that observed with fresh CB CD34+ cell isolation. The CD34+ cells were further cultured for up to two weeks (49 days) with identical combinations of cytokines with alemtuzumab 10 μg/ml. Secondary cultures without alemtuzumab were used as controls. A significantly higher percentage of CD34^+ ^cells (10.96 ± 5.09% vs. 2.24 ± 0.53%) was observed at day 42 (p < 0.05) and day 49 (2.34 ± 1.14% vs. 0.57 ± 0.39%, p < 0.05, Figure 5), respectively. At day 42, CD52+ cell population was 0.53 ± 0.31% with alemtuzumab vs. 5.59 ± 0.79% without alemtuzumab) and at day 49, CD52+ cells were 0.93 ± 0.55% with alemtuzumab vs. 16.56 ± 2.28% without alemtuzumab. Even at day 42 of culture, absolute CD34+ cells in cultures with alemtuzumab showed a 15% increase while the cultures without alemtuzumab showed a 76% decrease (absolute CD34+ expansion fold: 0.24, Figure 5B). By day 49, both cultures conditions showed a net loss of CD34+ cell numbers (absolute CD34+ numbers as percentage of original: 8% of original in control cultures vs 36% of original with alemtuzumab, Figure 5B).

## Discussion

The results from this study showed that the anti-CD52 antibody Alemtuzumab significantly enhanced *ex vivo *expansion of CB CD34^+ ^cells and TNC. In addition, compared to both the initiating cells and cytokines-alone control cultures, total CFU, especially CFU-GEMM and CFU-GM were expanded after treatment of alemtuzumab, and LTC-IC numbers preserved, confirming the retention of hematopoietic progenitors. Furthermore, the effects of alemtuzumab were more pronounced when the culture was extended for 49 days (14 days secondary cultures taken from CD34^+ ^cells selected out at day 35 of primary cultures). Although there is a net loss of CD34+ cell at day 49, the alemtuzumab did preserve more CD34+ cells compared to control cultures, which had loss of almost all CD34+ cells by day 49. This is likely because of the fact that differences in preserving progenitor/primitive cell populations become more prominent with longer term cultures, further confirming that alemtuzumab could potentially preserve primitive CD34+ hematopoietic cells.

Although the degree of expansion was modest, our experiments were aimed at comparing expansion with/without alemtuzumab, and were reasonable for this "early phase" cytokine combination, which was targeted at retaining immature stem cell populations rather than an overwhelming increase in absolute cell numbers. Immuno-homeostasis in *in vivo *transplantation/engraftment are far more complicated that just an *ex vivo *study and the results of *ex vivo *expansion caused by addition of alemtuzumab this study was indirect. As there was depletion of CD52 cells associated with the addition of alemtuzumab, future experiments will see if adding back CD52+ cells would abrogate the effect of alemtuzumab. Further optimization with the use of this drug in other cytokine combinations in large-scale expansion cultures is being carried out, including the SCID-repopulating assay.

One important issue in HSC expansion has always been the concern that expanding mature cell populations at the expense of the primitive progenitors could affect durable engraftment capacity [33]. The underlying mechanisms for alemtuzumab enhancement of *in vitro *cord blood expansion could be secondary to selective depletion of CD52^+ ^populations, such as lymphocytes and monocytes. Moreover, depletion of CD52^+ ^cell populations may result in removal of certain inhibitory cell populations, such as CD26+ lymphocytes and NK cells during *ex vivo *expansion of CB HSC [20,34]. The ability to retain the hematopoietic progenitors and stem cells in culture would enhance the likelihood of stable engraftment in recipients of these expanded products. Another potential risk of HSC expansion is the mature lymphocytes arising from these cultures, which could lead to a higher incidence of graft versus host disease (GVHD), thus negating a major benefit of CBT – which is the lowered incidence and severity of GVHD with increased tolerance towards HLA mismatches [3,35-37]. In our study, mature cells and lymphocytes were actively removed in culture with the maintenance of hematopoietic progenitors and stem cells, and a relative expansion of myeloid and megakaryocytic precursors; thus appearing to circumvent these two theoretical risks of expansion. Moreover, a recent study from Shah et al shows that alemtuzumab is effective in decreasing the incidence of GVHD without increasing the risk of relapse in pediatric patients [38]. Despite an initial proportional decrease in lymphocytes/monocytes, there was no subsequently absolute decrease of CD13^+^, CD14^+ ^and CD15^+ ^cell numbers, after 14 days of culture. Many clinical cord blood expansion trials now incorporate the expansion of one unit of cord blood following the infusion of another unexpanded unit in a double cord blood transplant setting. The unexpanded unit has, thus served successfully as a backup for potential loss of engraftment potential after cord blood expansion while the expanded unit serves to provide the first wave of myeloid recovery, thus decreasing the long engraftment period of CBT [39].

## Conclusion

In summary, our study shows that the use of alemtuzumab in *ex vivo *cord blood expansion results in an increase in total cell expansion while preserving hematopoietic CD34+ cell content. Furthermore, the expansion of myeloid and megakaryocytic precursors is also enhanced. This finding may contribute to current technologies in CB expansion and increase the availability of CBT for patients around the world.

## Methods

### Sample collection

CB samples were obtained from the Singapore Cord Blood Bank (SCBB) that failed to meet the banking criteria for storage. This study was approved by the Hospital's Ethics Committee. Further allocation of CBU used for this study was re-confirmed by the SCBB Research Advisory Ethics Committee. A total of nine CBU were used in this study. Three for CFU and LTC-IC study while six for the ex vivo expansion and flow analysis.

### CB CD34^+ ^cell isolation

Cord blood CD34^+ ^cells were isolated prior to expansion using a magnetic-cell sorting device (VarioMACS, Miltenyi, Germany). Briefly, mononuclear cells were fractionated by Ficoll-Hypaque^Plus ^(Amersham Pharmacia Biotech, Upsala, Sweden) density centrifugation. The CD34^+ ^cell fraction was then isolated using the direct CD34 isolation kit, LS columns and VarioMACS magnetic cell separator (Miltenyi Biotec, Germany) according to the manufacturer's instructions. The purity of the selected population was verified with anti-human CD34^+ ^antibody conjugated with phycoerythrin (PE, Anti-HPCA-1, Becton Dickinson, San Jose, USA) and analysed using the FACSCalibur flow cytometer and CellQuest Pro software (Becton Dickinson). Aldefluor assay was used to evaluate the activity of aldehyde dehydrogenase in the sorted CB CD34+ cells using the reagents from Stem Cell Technology (Stem Cell Technology, Vancouver, Canada) and the FACSCalibur flow cytometric analysis [30,32,40].

### Ex vivo expansion cultures of cord blood CD34^+ ^cells

10^5 ^CD34^+ ^cells isolated from same CBU were cultured in 2 ml StemSpan™ SFEM Medium (10%BSA, 10 μg/ml recombinant human insulin, 200 μg/ml human transferrin, 10^4^M 2-mercaptoethanol and 2 mM L-glutamine in Iscove's MDM, StemCell Technologies, Vancouver, Canada) supplemented with 50 ng/ml of stem cell factor (SCF, from Chemicon, USA), 50 ng/ml of thrombopoietin (TPO, from Chemicon, USA), 50 ng/ml of Flt-3 Ligand (Chemicon, USA), and 25 ng/ml of penicillin (10,000 uits/ml) and streptomycin (40 mg/ml, Gibco, USA). 10 μg/ml of alemtuzumab (Schering AG, Germany) was added with 10% of healthy human serum (collected from a consistent healthy adult donor with Ethics Committee approval) as a source of human complement. All cultures were performed at 37°C with humidified air containing 5% CO_2_. Culture medium was replenished on day 4, 7, 10, 14 with simultaneous harvest of small aliquots of cells for counts, phenotypic analysis, and culture assays. Trypan blue exclusion was used to determine cell viability. A total of six experiments were performed from six different CBU.

### Flow cytometry analysis

The effect of alemtuzumab on CD34+ stem cells populations after *ex-vivo *expansion of cord blood HSC was analyzed by flow cytometric analysis using a panel of monoclonal antibodies (mAbs), including CD34 and lineage specific mAbs against myeloid, erythroid, megakaryocytic and lymphoid lineages (CD13, CD14, CD90, CD41 and Glycophorin A, from Becton Dickinson, San Jose, USA and CD52, from Serotec, USA). Samples were incubated with mAbs at 4°C for 30 min, washed, fixed, acquired and analyzed using a FACSCalibur flow cytometer. At least 10,000 events were acquired for each analysis.

### Colony Forming Unit (CFU) assay

Colony assay was performed triplicate on cells obtained from day 0 (before culture) and day 14 after cultures. Cells were plated on 35 mm petri dishes (Nunc, Denmark) after mixing well with Methocult H4435 medium (1% methylcellulose in Iscove's MDM, 30% fetal bovine serum, 1% bovine serum albumin, 10^4 ^M 2-mercaptoethanol, 2 mM L-glutamine, 50 ng/ml SCF, 20 ng/ml, GM-CSF, 20 ng/ml IL-3, 20 ng/ml IL-6, 20 ng/ml G-CSF and 3 U/ml EPO, from Stem Cell Technologies, Vancouver, Canada). After incubation for 14 days at 37°C, granulocyte and macrophage colony forming units (CFU-GM), burst forming unit erythroid/erythroid colony forming units (BFU-E/CFU-E) and granulocyte-erythroid-macrophage-megakaryocyte colony forming units (CFU-GEMM) were enumerated according to manufacturer's guidelines under an inverted microscope (Axiovert 25, Carl Zeiss Pte Ltd, Singapore).

### Quantitative bulk culture assay of long-term culture-initiating cell (LTC-IC)

Bulk culture LTC-IC assay was established and maintained in triplicate following manufacturer's instructions (Stem Cell Technologies, Vancouver, Canada). Briefly, stromal layers were initiated with M2-10B4 murine fibroblast cell line (CRL1972) by seeding 1 × 10^6 ^cells in a 75 cm^2 ^culture flask in 15 ml RPMI (Gibco, Grand Island, NY, U.S.A) with 10% fetal calf serum (Hyclone, Logan, UT, USA). Half of the medium was changed weekly, and at log-phase growth, cells were trypsinized (Trypsin-EDTA; Invitrogen, U.S.A) and irradiated with an adsorbed dose of 8,000 cGy with a ^60^Co gamma irradiator (J.L. Shepherd & Associates, Canada). Feeder layers were then cultured in Long-term Culture Medium H5100 (12.5% horse serum, 12.5% FBS, 0.2 mM inositol, 20 mM folic acid, 10^4^M 2-mercaptoethanol, 2 mM L-glutamine in MEM- medium, from Stem Cell Technologies, Vancouver, Canada) supplemented with 10^6^mol/L hydrocortisone 21-hemisuccinate (Stem Cell Technologies, Vancouver, Canada). 24 hours later, freshly isolated day 0 CD34^+ ^cells and day 14 expanded cells were added in triplicates to irradiated M2-10B4 stromal layer. Cultures were maintained at 37°C 5% CO_2 _and 100% humidity for 5 weeks with 50% volume of medium changed weekly. At the end of the 5^th ^week, all the cells were harvested together (non-adherent cells were pipetted off and adherent cells were trypsinized) and fractions of cells were plated for CFU assay in H4435 medium as described above. After incubation for another 14 days at 37°C, total CFU numbers were counted and normalized against the initial cells. LTC-IC expansion was calculated from the LTC-IC derived total CFU numbers before and after culture under different conditions.

### Secondary culture for day 35 CD34^+ ^cells

To study the long term effects of alemtuzumab on hematopoietic progenitors and stem cells, cells were cultured for up to 35 days and CD34^+ ^cells were isolated using MACS from day 35 cultures with alemtuzumab as described above. The purity of CD34^+ ^cells was tested by flow cytometry analysis and selected CD34^+ ^cells were put into secondary culture with the same cytokine combination and 10% human serum, with or without 10 μg/ml alemtuzumab. After a further 7 to 14 days of culture, cells were harvested and analysis of CD34 and CD52 expression profiles was performed.

### Statistical analysis

Data was shown as mean ± SD. Group comparison for the statistical significance was calculated using Wilcoxon matched pairs nonparametric test. A p value of less than 0.05 was considered to be statistically significant.

## Competing interests

The authors declare that they have no competing interests.

## Authors' contributions

CKL was involved in designed, participated in all assay, analyzed data and drafted the manuscript. LS was involved in designed, flow cytometry and drafted the manuscript. QF was involved in CFU assay, LTC-IC assay and drafted the manuscript. PL was involved in critically evaluating and revising the manuscript. WTC and SNL were involved in sample process, cell cultures, Aldeflour assay and flow cytometry. WYKH was actively involved in concept design, coordination, interpretation of data, drafting and critically revising the manuscript. All authors read and approved the final manuscript.
